# Elucidating mechanisms of sunitinib resistance in renal cancer: an integrated pathological-molecular analysis

**DOI:** 10.18632/oncotarget.23163

**Published:** 2017-12-08

**Authors:** Henriett Butz, Qiang Ding, Roy Nofech-Mozes, Zsuzsanna Lichner, Heyu Ni, George M. Yousef

**Affiliations:** ^1^ Department of Laboratory Medicine, and The Keenan Research Centre for Biomedical Science of St. Michael's Hospital, Toronto, ON, M5B 1W8, Canada; ^2^ Department of Laboratory Medicine and Pathobiology, University of Toronto, Toronto, ON, M5S 1A8, Canada

**Keywords:** sunitinib, resistance, miRNAs, renal cell carcinoma, metastasis

## Abstract

Upon sunitinib treatment of metastatic renal cell carcinoma patients eventually acquire resistance. Our aim was to investigate microRNAs behind sunitinib resistance.

We developed an *in vivo* xenograft and an *in vitro* model and compared morphological, immunhistochemical, transcriptomical and miRNome data changes during sunitinib response and resistance by performing next-generation mRNA and miRNA sequencing. Complex bioinformatics (pathway, BioFunction and network) analysis were performed. Results were validated by *in vitro* functional assays.

Our morphological, immunhistochemical, transcriptomical and miRNome data all pointed out that during sunitinib resistance tumor cells changed to migratory phenotype. We identified the downregulated miR-1 and miR-663a targeting FRAS1 (Fraser Extracellular Matrix Complex Subunit 1) and MDGA1 (MAM Domain Containing Glycosylphosphatidylinositol Anchor 1) in resistant tumors. We proved firstly miR-1-FRAS1 and miR-663a-MDGA1 interactions. We found that MDGA1 knockdown decreased renal cancer cell migration and proliferation similarly to restoration of levels of miR-1 and miR-663.

Our results support the central role of cell migration as an adaptive mechanism to secure tumor survival behind sunitinib resistance. MDGA1, FRAS1 or the targeting miRNAs can be potential adjuvant therapeutic targets, through inhibition of cancer cell migration, thus eliminating the development of resistance and metastasis.

## INTRODUCTION

Renal cell carcinoma (RCC) is the most frequent malignancy of the adult kidney and its incidence has been steadily rising by 2–4% each year [1]. Up to 30% of patients present with metastases at diagnosis [2]. Patients with metastatic disease have 13 months median survival and a 5-year survival rate under 10% [3]. RCC is considered to be highly resistant to conventional therapeutic modalities including chemotherapy and radiation [4, 5]. It is highly vascularized cancer due to the hypoxia induced factor (HIF) stabilization as a consequence of VHL inactivation [6]. HIF accumulation leads to transactivation of molecules involved in angiogenesis including VEGF and PDGF [7]. The angiogenesis-inhibitor sunitinib is the first-line therapy for metastatic RCC. This small molecule multi-tyrosine kinase inhibitor [4] inhibits VEGFR-1, -2, -3, PDGFR-α and PDGFR-β, Fms-like tyrosine kinase 3 (FLT3), stem cell factor receptor (KIT), colony stimulating factor receptor type 1 receptor; and the glial cell line–derived neurotrophic factor receptor [8, 9]. Sunitinib treatment prolonged both progression-free and overall survival [10] and response rates to sunitinib were higher compared to interferon [11, 12].

Approximately 70% of patients show initial response to therapy but unfortunately in 6-12 months patients eventually acquire resistance and disease progression [13–15]. Potential predictive markers for sunitinb treatment have been suggested, but none is in clinical practice [16]. The remaining 30% are refractory to therapy (primary or intrinsic resistance) [15]. The causes behind resistance are not fully understood. A number of mechanisms have been proposed [17, 18]. Recent data suggest that resistance mechanisms include restoration of angiogenesis (through VEGF-independent pathways) and reduced bioavailability (increased efflux or lysosomal sequestration) [18]. Vasuclar co-option was also highlighted in other cancers [19]. Interestingly, current evidence also shows that TKI resistance is reversible [20, 21].

Recent literature suggested that miRNAs are involved in RCC pathogenesis (13), and the aqusition of aggressive behavior [22, 23], and their clinical utility as RCC biomarkers and therapeutic targets was also highlighted [24].

In the current study we developped an *in vivo* xenograft and *in vitro* models for sunitinib resistance. We performed next-generation RNA and miRNA sequencing of control, sunitinib-sensitive and resistant xenograft tumors. We aimed to find potential mechanisms and investigate the roles of miRNAs in sunitinib resistance.

## RESULTS

### Characterization of sunitinib sensitive and resistant xenograft RCC tumors

**Figure 1:**
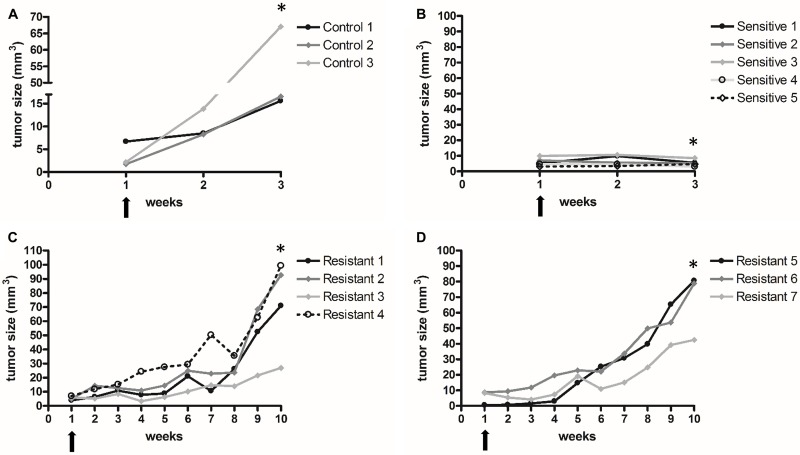
Vehicle or sunitinib treatment in control (**A**) sensitive (**B**) and resistant (**C**–**D**) xenograft groups. Arrows show treatment start points. Mice were sacrified at the time points indicated by stars [^*^]. Sunitinib treatment resulted in stability of tumor volume for 3 weeks (no tumor regrowth) compared to control group. Resistance started to develop after four weeks of treatment and tumors grew rapidly after 6-7 weeks.

**Figure 2:**
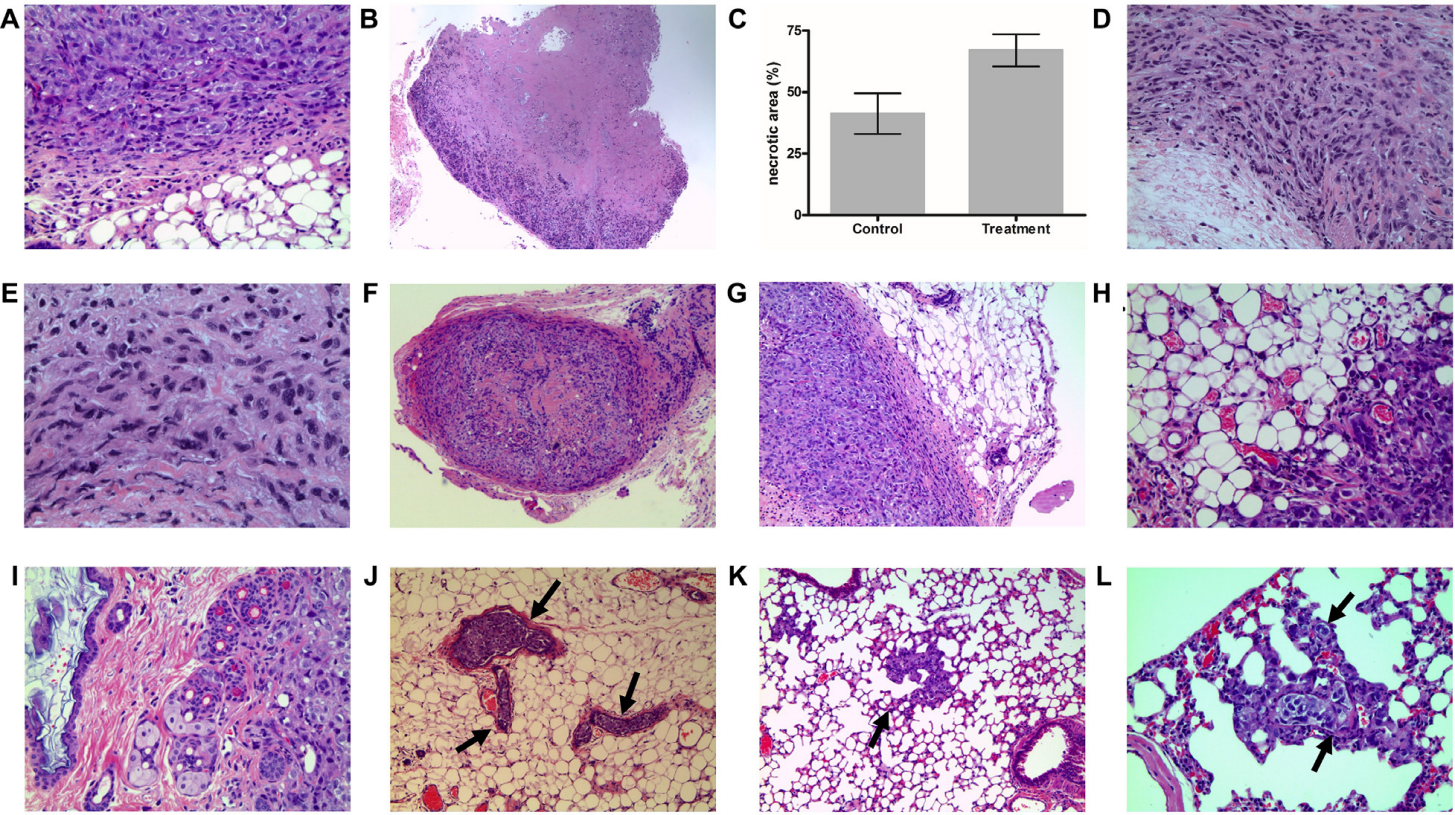
Xenograft tumor tissue H&E staining (**A**) Pretreatment tumors showed classic RCC morphology with rounded cells and occasional clearing of cytoplasm. (**B**) Large areas of necrosis were observed in sunitinib-sensitive tumor. (**C**) A bar graph comparing percentage areas of necorsis in sunitinib-treated vs. control xenografts. (**D**) Sarcomatoid phenotype with spindle cells was observed in resistant tumors. (**E**) Poorly differentiated cells were also seen in resistant tumors. (**F**) Low power magnification showing sensitive tumor with well-circumscribed border, fibrosis and chronic inflammatory infilitrate in the capsule. (**G**) Higher magnification showing pushing borders with fibrosis and inflammation in sunitinib-sensitive tumors. (**H**) Irregular infiltrative tumor margins with fat invasion were seen in sunitinib-resistant tumors (**I**) Resistant tumors were more aggressive with invasion to adjacent organs (skin dermis in this case). (**J**) Resistant tumors showed extensive vascular invasion (vessels indicated by arrows). (**K**) Lung metastasis in resistant tumors (arrow). Metatatsis showed vascular co-option pattern. (**L**) Vascular-co-option (arrow) in resistant tumor metastasis. Tumors travel along blood vessels of the wall of the alveoli while preserving the alveolar spaces.

Mice were subcutaneously injected with human renal cell carcinoma cell lines. After tumor formation, animals received sunitinib treatment, as described in the materials section. RECIST criteria was used to assess reponse to treatment. Sunitinib treatment resulted in reduction/stability of tumor volume for about 4 weeks (Figure 1). Resistance started to develop after four weeks of treatment and tumors grew rapidly after 6-7 weeks (Figure 1). On microscopic examintaion, tumors xenografts showed the classic RCC morphology (Figure 2A).

As expected, when comparing tumors of the same size, there were significant differences in tumor characteristics detailed in Table 1, there were significantly larger necrotic areas in sunitinb-treated tumors compared to the untreated controls (Figure 2B-C). In sunitinib-resistant tumors, necrotic areas were also seen (consistant with earlier treatment effect). Interestingly, resistant tumors exhibited focal sarcomatoid phenotype with spindle cells (Figure 2D) and also poorly differentiated cells (Figure 2E) compared to controls and sensitive tumors.

**Table 1:** Semi-quantitative morphological analysis of xenograft tumors

	786O xenograft Untreated control	786O xenograft Sunitinib sensitive	786O xenograft Sunitinib resistant
Necrosis Area	++	++++	+++
Sarcomatoid change/poor differentiation	–	–	+
Border	Well/regular	Infiltrative/regular	Infiltrative/irregular
Invasion to adjacent	–	–	+
Vascular invasion	–	–	++++
Distant Metastasis	–	–	++
Vascular co-option	–	–	++

When comparing sensitive to resistant tumors, sensitive tumors showed well-circumscribed border with fibrous capsule with inflammatrory infiltrate (Figure 2F–2G). Resistant tumors, on the other hand, had infiltrative irrelgular borders with invasion to surrounding adipose tissue. (Figure 2H). Moreover, sunitinb-resistant tumors had a much more aggressive behaviour, manifested by invasion to adjacent organs (Figure 2I) to In addition to the sarcomatoid differentiation and local invasion, resistant tumors showed more aggressive behaviour demonstrated by the presence of extensive vascular invasion (Figure 2J), and liver and lung metastases (Figure 2K). No metatsis were observed in sunitinb-sensitive tumors.

Sunitinb-resistant tumors exhibited an interesting pattern of vascular co-option [19]. This was characterized by the migration of tumor cells along blood vessels of the alveolar walls of the lung without destruction of the alveolar spaces (Figure 2K–2L) [19]. This phenomenon was described before as a mechanism for tumor cells to escape antiangiogenic treatment effect by migrating through and maintaining normal architecture to be able to utilize the host's normal vasculature (capillaries) for oxygen and nutrition. To our knowledge, this was not previously reported in kidney cancer.

**Figure 3:**
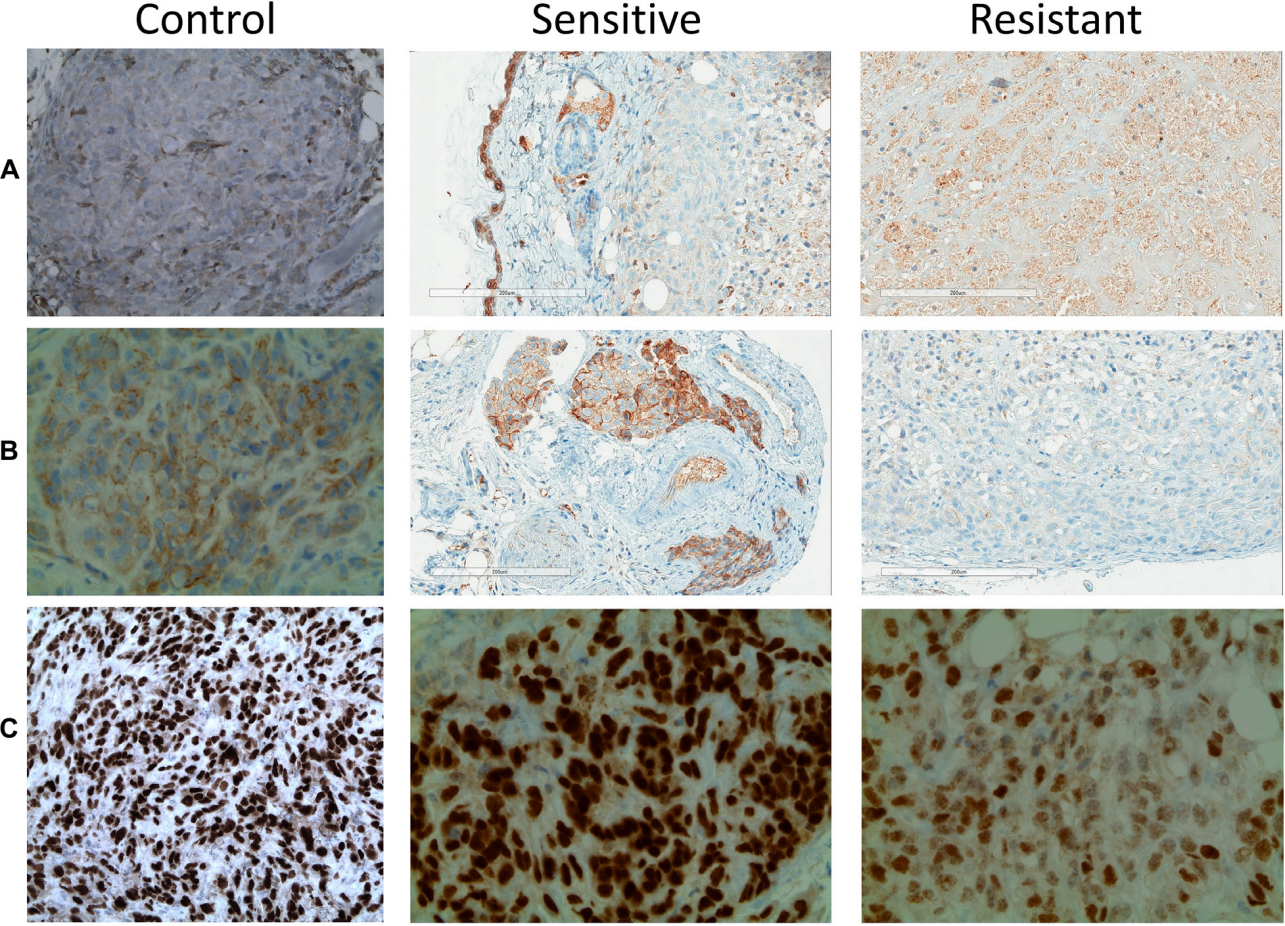
Immunostaining patterns of sunitinib sensitive and resistant tumors (**A**) COX-2 is increased in resistant tumors compared to control and sensitive ones. E-cadherin (**B**) and PAX8 (**C**) showed reduced staining in resistant tumors compared with control and sensitive tumors.

Based on previous literature data regarding TKI resistance we investigated COX2 and PAX8 immunostaining on resistance xenograft tumors [9, 25, 26]. We found slight COX2 expression increase is in resistant tumors (Figure 3A), which is in line with previous reports showing that COX2 inhibition was able to potentiate sunitinib effect in RCC xenografts [9]. There was also a significant reduction of E-cadherin expression in resistant compared to sunitinib-sensitive tumors, indicating the development of an EMT phenotype (Figure 3B). PAX8 is a cell lineage restricted transcription factors essential for embryonic kidney development [26]. It is used in as a marker of renal differentiation. PAX8 expression was decreased in resistant tumors (Figure 3C), indicating the same trend towards dedifferentiation and the development of an epthelial to mesenchymal phenotype (EMT).

### Differential gene and miRNA expression between sunitinib-sensitive and resistant xenograft RCC tumors

In order to obtain an insight about the biological attributes of sunitinib response and resistance, we compared the transcriptomic profiles (mRNA and miRNA) of control, sensitive and resistant tumors using next generation sequencing. Cluster analysis based on mRNA expression data showed that resistant tumors are separated from sunitinib-sensitive and control groups (Supplementary Figure 2A–2B). Similar results were obtained with miRNA clustering analysis (Supplementary Figure 2C–2D).

Interestingly, there were minimal significant molecular differences between sensitive tumors and controls (only 52 mRNAs and no miRNAs) (Supplementary Figure 2B, 2D). Comparing these two groups by BioFunction Analysis, we identified “proliferation of endothelial cells”, “vasculogenesis”, “growth of epithelial tissue” and “migration of cells” to be down-regulated in sunitinib-sensitive tumor grafts (Supplementary Table 1). This is in keeping with the reported antiangiogentic role of typroisne kinase inhibitors.

**Figure 4:**
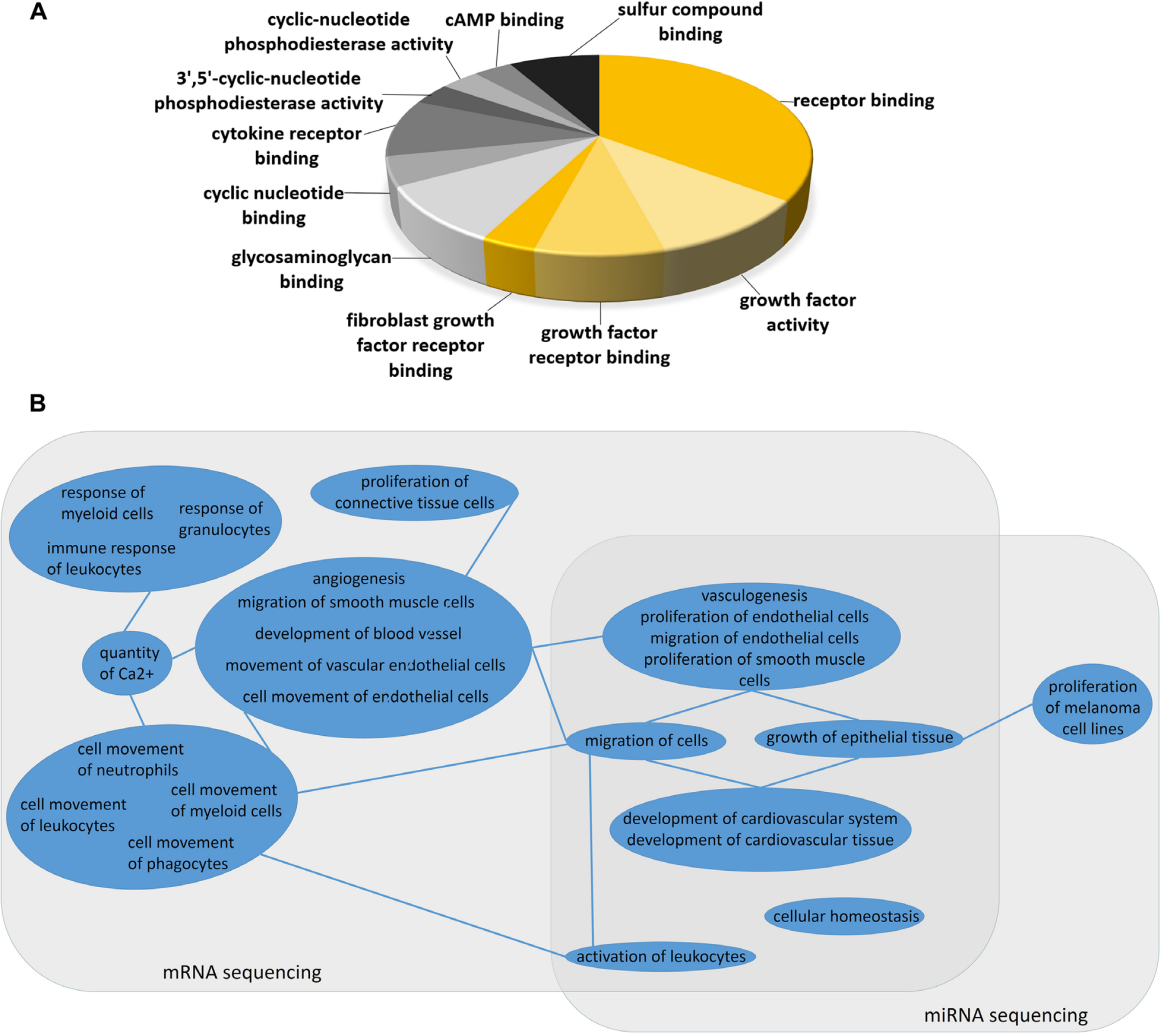
(**A**) Molecular Function Analysis of miRNA changes in sunitinib resistant tumors. (**B**) Comparison of Diseases and BioFunction analysis of mRNA and miRNA sequencing between sunitinib resistant and sensitive xenograft tumors. BioFunction Terms are grouped by similar biological role (blue circles). Lines among similar BioFunction groups indicate connections in function. The clustered BioFunctions altered in mRNA expression profiling related to angiogenesis, immune response, cell movement were all significant in network with calcium signaling and cell proliferation. Other BioFunction terms related to vasculogenesis, cell migration and leukocyte activation were significant in both mRNA and miRNA sequencing results.

We next compared sunitinib-resistant to sensitive tumors, using Comparative Pathway Analysis for mRNA sequencing data and for tissue specific-target predicted miRNAs (TSTP miNRAs) to increase biological relevance of miRNA function. Genes related to cell migration, regulation of the Epithelial-Mesencymal Transition (EMT) Pathways, and human embryonic stem cell pluripotency were altered significantly in resistant tumors. Additionally, pathways related to immune response and inflammation was dysregulated between the two groups (Supplementary Table 2). These findings match with the differential inflammtoary response morphology that was observed [27]. BioFunction and Gene Ontology Analysis showed very comparable results (Supplementary Table 2). Pathway analysis of miRNA expression differences in sunitinib-resistant to sensitive tumors showed strikingly similar result to pathway analysis of altered mRNA profile in the same samples including “Epithelial-Mesencymal Transition”, “Human Embryonic Stem Cell Pluripotency” and signaling related to immune response. Molecular Functions related to growth factor receptors represented a considerable proportion of changed molecular functions regulated by miRNAs in sunitinib resistant tumors (Figure 4A). Next, we compared the alterations in Diseases and BioFunctions analysis between mRNA and miRNA sequencing, and found strong resemblance between the relevance of the two levels of transcript regulation (Figure 4B) indicating the role of miRNAs in sunitinib resistancy.

### miRNAs are involved in sunitinib resistance *in vitro*

**Figure 5:**
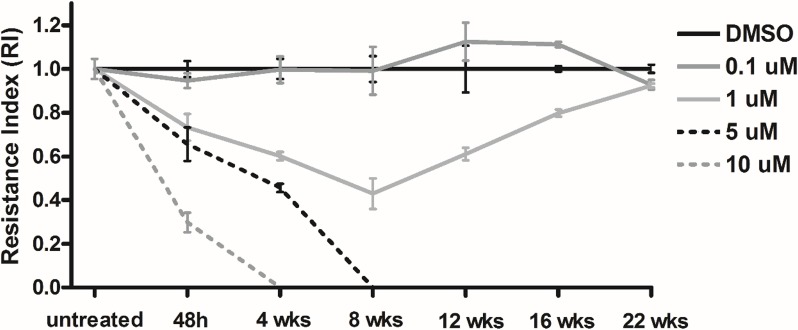
*In vitro* sunitinib response and resistance in RCC cell lines Different concentrations of sunitinib were tested and a concentration of 1 μM resulted in initial response followed by resistance after 8 weeks. Resistance index (RI) was calculated as previously described by Pénzváltó et al, 2013.

We developped an *in vitro* sunitinib resistance model to study the functional contribution of miRNAs to resistance. After optimization of sunitinib concentration (as detailed in the materials section), we used effective sunitinib concentration of 1 μM. Cells showed initial response and then developed resistance after 8 weeks of sunitinib treatment. After 16 weeks cells reached the original growth rate (Figure 5). We compared miRNA expression profile between cells treated for 48 hours (sensitive) and for 23 weeks (resistant). miRNA alterations *in vitro* were cross-matched to our *in vivo* results (discussed above) and expectedly, comaparable results were obtained (Table 2). Performing Pathway and BioFunction analysis, we identified almost identical pathways and cellular processes through miRNA and gene expression analyses of the xenograft experiment (Supplementary Table 3).

**Table 2:** Differentially expressed miRNAs between sunitinb-sensitive and resistant tumors^*^

miRNA name	*in vitro* (log2FC)	*in vivo* (log2FC)	adj.P.Val
hsa-miR-483-5p	−8.5	−6.9	0.00000
hsa-miR-483-3p	−3.2	−4.5	0.00000
hsa-miR-18a-3p	−10.6	−2.3	0.00023
hsa-miR-618	−1.2	−1.7	0.03615
hsa-miR-486-3p	−1.8	−1.6	0.01404
hsa-miR-1254	−1.1	−1.5	0.01257
hsa-miR-1247-5p	−2.5	−1.4	0.01087
hsa-miR-875-5p	−16.1	−1.4	0.03615
hsa-miR-939-5p	−2.4	−1.4	0.03615
hsa-miR-7-1-3p	−1.1	−1.4	0.00452
hsa-miR-454-5p	−1.6	−1.2	0.00356
hsa-miR-1276	−8.9	−1.2	0.03128
hsa-miR-543	−3.7	−1.2	0.03615
hsa-miR-30e-3p	−2.1	−1.2	0.00066
hsa-miR-30c-2-3p	−2.8	−1.1	0.00042
hsa-miR-29c-5p	1.3	2.0	0.03128
hsa-miR-655-3p	9.8	2.2	0.00131
hsa-miR-145-5p	16.4	2.2	0.00011
hsa-miR-10b-5p	1.1	2.3	0.00001
hsa-miR-615-3p	1.6	2.7	0.00000
hsa-miR-9-5p	1.2	2.8	0.00218
hsa-miR-22-5p	1.5	3.0	0.00001
hsa-miR-15a-5p	4.7	3.2	0.00000
hsa-miR-758-3p	3.6	3.7	0.00001
hsa-miR-382-5p	5.3	4.0	0.00016
hsa-miR-183-3p	5.2	4.2	0.00002
hsa-miR-223-3p	8.7	4.2	0.00000
hsa-miR-335-3p	2.1	4.3	0.00000
hsa-miR-582-5p	8.4	4.5	0.00031
hsa-miR-154-5p	1.1	4.5	0.00000
hsa-miR-190a-5p	4.5	5.0	0.00000
hsa-miR-375	30.8	5.1	0.00000
hsa-miR-769-5p	12.1	7.0	0.00000
hsa-miR-889-3p	2.6	7.1	0.00000
hsa-miR-654-3p	5.8	7.5	0.00000
hsa-miR-92a-3p	12.4	9.2	0.00000

### Network analysis

In order to thoroughly investigate the role of the gene-miRNA axis of interaction in resistance, we integrated gene and miRNA expression data into an interaction network that is filtered to kidney-related miRNAs and mRNAs (Supplementary Figure 2E). Based on network structure and literature data, we identified miR-663a (downregulated in resistance) and MDGA1 (overexpressed in resistance) as “hubs” (HUBs are defined as the top 10% of the nodes [genes or miRNAs] with highest number of interaction) that can play a central role in resistance (Supplementary Table 4). Additionally, MDGA1 has been also shown to increase cell motility [28]. miR-663a is predicted to target MDGA1 and is reported in literature to induce cell differentiation and suppress tumor growth, invasiveness and cellular migration in multiple cancer types [29–31]. Our network also highlighted FRAS1 and miR-1. FRAS1 is predicted target of miR-1 and is reported to be involved cell migration and invasion [32]. All four molecules are involved in cell motility and migration which is in line with our *in vivo* and *in vitro* results [33, 34], therefore we selected miR-1-FRAS1 and miR-663a-MDGA1 interaction for further investigation on RCC cell functions.

### Validation of the effect of miR-1-FRAS1 and miR-663a-MDGA1 interactions on cell behaviour

**Figure 6:**
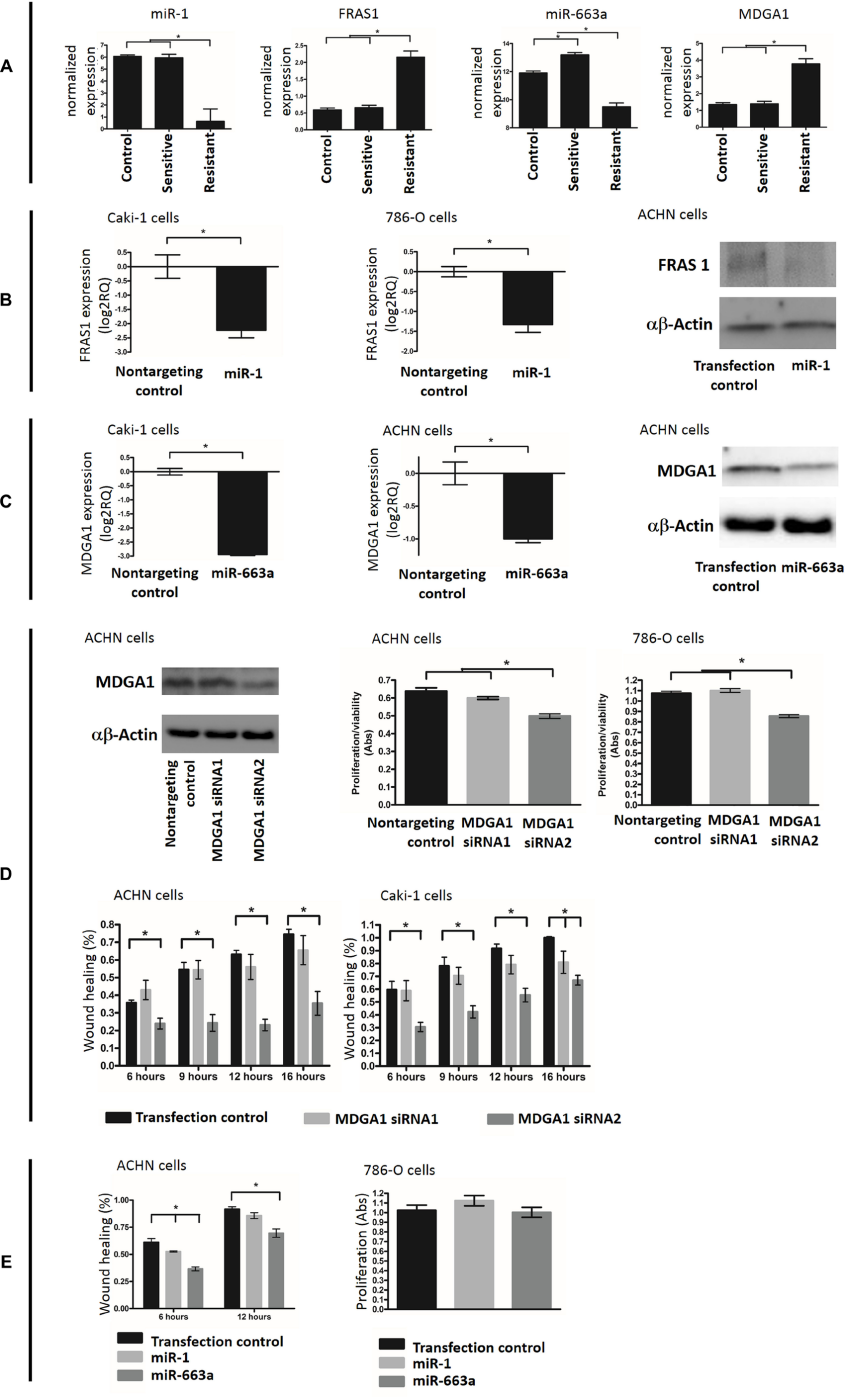
(**A**) Expression of miR-1, FRAS1, miR-663a and MDGA1 in control, sunitinib sensitive and resistant xenografts. (**B**) Experimental validation of miR-1-FRAS1 interaction. miR-1 overexpression was able to significanly reduce the level of FRAS1 at both mRNA (measured by qRT-PCR, the two left graphs) and protein (assessed by western blot, the right graph) levels. (**C**) Experimental validation of the ability of miR-663a to target MDGA1. miR-663a over expression was able to significanly reduce the level of MDGA1 mRNA (qRT-PCR analysis, left) and protein (western blot analysis, right). (**D**) siRNA resulted in decreased MDGA1 protein expression (western blot). MDGA1 knock-down resulted in significant reduction of the rate of cell proliferation and migration (wound healing assay). (**E**) Transfection of miR-1 and miR-663a mimics led to decreased cell migration but had no effect on proliferation.

Our data showed inverse correlation in expression levels between miR-1 and FRAS1 and between miR-663 vs MDGA1 (Figure 6A), indicating that the later genes are potential targets for the former miRNAs. We also found differential expression with maintained inverse correlation between these molecules in resistant compared to sunitinib-sensitive xenografts, indicating potential involvement in resistance (Figure 6A). We then experimentally validated these miRNA-gene interactions in cell line models. miR-1 and miR-663 mimics transfection resulted in reduction of the expression levels of FRAS1 and MDGA1, respectively at both the protein and mRNA levels (Figure 6B–6C).

We then examined the effect of these molecules on cancer cell behavior. Loss-of-function experiments were done using MDGA1-specific siRNAs. MDGA1 knockdown resulted in decreased migration and proliferation in ACHN, 786-O and Caki-2 kidney cancer cell lines (Figure 6D). Gain of fucntion experiments were done through restoring the downregulated miR-1 and miR-663 by miRNA mimics transfection which also resulted in decreased cell migration (Figure 6E). However we could not detect any effect of miRNA mimics on viability/proliferation (Figure 6E).

Taken together, our results show a multifactorial mechanism that results in resistance and support a central role of cell migration as an adaptive mechanism, which is mediated, at least in part, through a miRNA-target network of interaction. Resistant tumor cells acquire migratory phenotype and migrate to host vessels (vascular co-option) to overcome angiogenesis inhibition by TKIs.

## DISCUSSION

Sunitinib resistance is a significant problem in treatment of metastatic RCC. We showed, through morphological, immunohistochemical and molecular analyses that changing to migratory phenotype play a role in resistance, and that resistant tumors acquire a more aggressive phenotype with metatstic potential. This represents a cornerstone for future research to identify adjuvant therapy to prevent or slow resistance. We found that PAX8 is decreased in resistant tumors. PAX8 is a cell lineage restricted transcription factor essential for embryonic kidney development [26]. It is expressed in renal tubular cells in fetal and adult kidneys and RCC [26]. PAX8 is also involved in the regulation of L1-CAM, an important therapeutic and prognostic protein in RCC [6]. It was previoulsy shown that PAX8 downregulation resulted in increased L1-CAM expression and enhanced migration of kidney cancer cells [6]. Therefore PAX8 downregulation in sunitinib resistant cells could be a part of an evolving migratory phenotye. The role of PAX8 is, however, yet to be further investigated. Others reported increased PAX8 expression in sarcomatoid RCC and metastases [35–37]. Besides, we found decreased E-cadherine in sunitinib-resistant tumors, again confirming a migratory phenotype as loss of E-cadherin is considered to be a basic event in EMT.

It has been shown that drug resistance is reversible in RCC [20]. Our mRNA and miRNA transcription profiling showed no evidence of angiogenesis rebound (meaning that sunitinib still exerts its effect on growth factor receptors) in resistant tumors. Recent literature showed that TKI resistant cells exhibit vessel-cooption through increased migration [19]. Instead, pathways, biofunctions and GO categories of both mRNA and miRNA profiles pointed to cell migration. In both mRNA and miRNA sequencing analysis we found immune response and immune cell activation appeared. Interestingly angiogenesis is regulated especially in cell movement/migration aspect that was indicated by mRNA changes. The proliferative aspect of angiogenesis (proliferation if endothelial and smooth muscle cells) is likely controlled by miRNAs as it occurred in both miRNA and mRNA level. MiRNA expression changes are supported by our *in vitro* model which showed comparable changes.

Network analysis rely on individual molecular interactions, making them unbiased and advantageous over other system biology approaches like pathway, BioFunction or GO, for giving a better insight on the function of individual molecules.

We identified two underexpressed miRNAs; miR-663 and miR-1, targeting MDGA1 and FRAS1, respectively. FRAS1 is involed in cell adhesion and it was previously reported that FRAS1 knockdown reduces A549 lung cancer cell migration and invasion through downregulation of focal adhesion signaling [32]. The role of MDGA1 protein was related to cell adhesion molecules implying MDGA1 involvement in cell migration and adhesion [28]. Its expression led to increased cell motility and cell-cell adhesion and reduced adhesion to extracellular matrix proteins in MDCK canine kidney cells [28]. Our data is the first to experimentally indicate miRNA regulation of either molecule. We firstly demonstrated that FRAS1 and MDGA1 were regulated by miR-1 and miR-663a, with subsequent potential role in sunitinib resistance through regulating cell migration.

miR-1 is a tumor suppressor miRNA that is downregulated in several cancers [33]. It targets a number of molecules including MET, Slug, CCND2, CXCR4 [33]. It has been shown that miR-663 inhibits tumor cell migration and invasion of thyroid carcinoma and glioblastoma cells [30, 31]. Interestingly, beside tumor cells it inhibits migration of endothelial and vascular smooth muscle cells as well [38, 39]. This is of particular importance in RCC, which is a highly vascular tumor. The cause behind downregulation of miR-663 in resistant tumors still needs to be elucidated but studies suggeted increased promoter methylation in leukemic cells [40, 41] which would correspond the findings that sunitinib resistance could be reversible [20] through methylation being a reversible epigenetic change.

Our findings are similar to a mechanism that was recently suggested for sorafenib resistance in hepatocellular carcinoma, namely, cell invasion mediated vessel co-option [19]. We found that resistant tumor cells acquire a migratory phenotype in the lack of blood supply, and through invading the host vessels they develop metastases. This is supported by other's findigs that highly angiogenic primary ccRCC tumours can give rise to non-angiogenic metastases, which may be resistant to antiangiogenic therapy [40].

In conclusion, our phenotypic, immunhistochemical, mRNA and miRNA results support a central role of cell migration as an adapting mechanism. Tumor cells (instead of rebound angiogenesis) aquire a migratory phenotype and migrate to vessels (specifically to spots where there is more blood supply e.g. lung). Our hypothesis is that “changing to migratory phenotype” can be a mechanism of sunitinib resistance that can secure tumor cell survival. We identified the role of MDGA1 and FRAS1 regulated by miR-663a and miR-1 in this process. They can be potential adjuvant therapeutical targets. By inhibiting them it would be possible to inhibit migration hence slow or delay metastasis development.

## MATERIALS AND METHODS

### Xenograft experiment

Female NOD/SCID/γ mice were used for sunitinib resistance modelling. Animal procedures were done according to the institutional animal care guidelines. 5-week-old mice were subcutaneously injected with 786-O human RCC cell line (ATCC CRL-1932), 1.5 × 10^6^ cells in both flanks. Tumor size was measured twice per week using calipers, and tumor volume was calculated as width^2^ × lenght × 0.5. After tumor formation (∼1 week), animals received oral gavage of sunitinib (purchased from Selleckchem, USA) as a citrate-buffered (pH 3.5) solution daily (7 days a week) at the dosage of 40 mg/kg or vehicle. Treatment response was assessed accourding to the RECIST criteria. Mice were sacrificed at the following timepoints: vehicle control (*N* = 4), sunitinib-sensitive (3 weeks of treatment, with response and before signs of resistance) (*N* = 5), and the resistant group (10 weeks of treatment, with resistance after initial resposne) (*N* = 7). Upon autopsy, xenograft tumors were stored in RNA later solution (Thermofisher Scientific, AM7021) until further use. Kidneys, livers and lungs were also collected for formalin fixation and immunhistochemical analysis.

### *In vitro* sunitinib resistance model

We investigated effect of different sunitinib concentrations on inducing apoptosis of RCC cell lines by Annexin V-Propidium Iodide flow cytometry (Supplementary Figure 1). At 0.1 μM sunitinib concentration that is considered to be pharmaclogically relevant we could not detect significant effect in line with other publications [20, 42]. Different concentrations were tested and and at sunitinib concentration of 1 μM, cells showed initial response and then cells developed resistance after 8 weeks of treatment.

### Annexin V and propidium iodide flow cytometry

Annexin V-FITC (AV) and Propidium Idoide (PI) were purchased from BD (cat.^#^: 556420, 556463). All steps were performed according to the manufacturer's instructions. After 24 h 0.1, 1.0, 5.0, 10.0 μM sunitinib treatment ACHN cells were harvested and suspended in binding buffer and count by Vi-Cell (Beckman). Annexin V assay was performed by staining with Annexin V-FITC for 15 min at 4°C in dark. Then stained cells were suspended in binding buffer and were analysed by flow cytometry after adding PI on a MACS Quant flow cytometer (Miltenyi Biotec, Auburn, CA, USA) at an event rate of < 500 evs/s. Data was dissected using Multicycle AV (FCS Express, DeNovo Software, Los Angeles, CA, USA). Intact cells are characterized by AV–/PI–, early apoptosis by AV+/PI–, apoptosis by AV+/PI+ and necrotic/dead cells by AV–/PI– [43].

### Viability/proliferation and migration assays

Cell proliferation/viability was controlled by WST-1 cell proliferation reagent (Roche Applied Science) as previously described [44]. Cell migration was investigated by wound healing assay as described before [44, 45].

### RNA extraction

Total RNA extraction from cells and xenograft tissues for RT-qPCR and next generation sequencing was performed using miRNeasy kit (^#^217004, Qiagen, Mississauga, Canada), as in our previous publications [46]. RNA concentration and quality were asessed using NanoDrop (Thermo Scientific, Hudson, NH, USA).

### Next generation sequencing

For *miRNA sequencing*, libraries were prepared using the Truseq Small RNA Library Sample prep kit (Illumina Inc., San Diego, CA, USA Cat ^#^RS-200-0012). MiRNA was then precipitated, followed by adaptor ligation, cDNA synthesis, and amplification. and rapid run sequencing of 1 × 51 cycles (Illumina Inc., San Diego, CA, USA Cat ^#^FC-402-4002), as described in the Supplemetary Methods. Reads were aligned to a database of mature RNA sequences (mirBase 20) using novoalign v2.08.02. The number of reads uniquely mapping to each mature RNA sequence were counted. Raw counts were normalized and transformed using the R package EdgeR and the voom transformation from the R package limma. Differential expression between groups was determined using the limma package.

For *RNA sequencing*, libraries were prepared using the TruSeq Stranded mRNA Sample Prep Kit (Illumina, Cat ^#^RS-122-2101). Paired-end cluster generation (Illumina Inc., San Diego, CA, USA Cat ^#^PE-401-3001) and sequencing of 2 × 101 cycles (Illumina Inc., San Diego, CA, USA Cat ^#^FC-401-3001) was performed for all libraries on the Illumina Hi-Seq 2000/2500 platforms (Illumina Inc., San Diego, CA, USA), as detailed in our supplementary data. Paired end reads were trimmed to 75bp. Reads were aligned to the UCSC hg19 reference genome using tophat (v2.0.3), followed by transcript assembly and estimation of expression levels (fpkm) with cufflinks (v2.0.2). Differential expression between groups was determined using the cuffdiff module of cufflinks, providing fold change estimates, associated *p*-values and q-values. The cufdiff output was reviewed with the R package cummeRbund, to generate a variety of visualizations of the data and summaries of the most significant differently expressed events.

### Bioinformatics analysis

*Pathway analysis and tissue-specific target prediction.* We used Ingenuity Pathway Analysis (IPA) for pathway and comparative analysis as previously described [44] to investigate the possible biological relevance of gene and miRNA expression changes in xenograft tissues and cell culture experiments (Ingenuity Systems, Redwood City, CA, USA). To identify expression alterations fold change filter was set to 2-fold, and then unpaired *t*-test was used to identify significant (*p* < 0.05) gene expression changes with multiple testing correction (Benjamini-Hochberg) in order to get better false discovery rate. miRNA targets were identified using TargetScan, miRecords, TarBase and Ingenuity Knowledge Base (IPAKB). We performed “tissue-specific target prediction” using a multistep filtration procedure as described before [44]. Briefly, using significantly deregulated miRNAs and mRNAs we selected miRNA-mRNA pairs by target predictions combined with the presence of inverse expression alterations.

### Biofunction and Gene Ontology (GO) analysis

Biofunctions were also determined by IPA gene set enrichment analysis and results were characterized by activation/inhibition Z-scores derived from gene/miRNA expression. GO categories of miRNA target genes were determined Ingenuity by using Generic Gene Ontology (GO) Term Mapper.

### Network formation and structure analysis

In order to identify the role of genes and miRNAs in our sunitinib resistance model we generated a miNA-target gene network. Ingenuity Knowledge Base (IPAKB) was used to discover interaction between genes and between genes and miRNAs based on known litearure data. We visualized and analyzed network structure using Cytoscape 3.1.0 software. We indicated mapping node's colour and size by node degree as described previously [22].

### miRNA expression profiling and qRT-PCR miRNA and gene expression

We determined miRNA expression profile alteration in cell culture by TaqMan Array Human MicroRNA Card Set v3.0 (Thermofisher Scientific, Cat^#^: 4444913) containing assays for a total 754 of human microRNAs as described before [28]. Individual miRNA expression was determined on the Viia 7 Real-Time PCR System (Life Technologies) [27] using the individual TaqMan assays for miR-1 (Assay ID: 000385).

### siRNA and miRNA mimics transfection

The primary 786-O and metastatic ACHN and Caki-2 kidney cancer cell lines were purchased from American Type Culture Collection. Cells were transfected with 1 of 2 different Locked Nucleic Acid^®^ small interfering RNAs (siRNAs) as previously described [44] against MDGA1 siRNA1 Cat 4392420^#^ ID s49009) (100 nM) (Life Technologies), or siRNA2 Cat 4392420^#^ ID s49011 (100 nM) using Lipofectamine RNAiMAX (Life Technologies). Gene knockdown was verified on protein level by western blot using Santa Cruz Bio antibody (Cat^#^ sc-168569 MDGA antibody).

Primary 786-O and metastatic ACHN and Caki-2 kidney cancer cell lines were transfected with 60 and 120 nM any of miR-1 (ID: MC10617, miR-663a miRNA Mimics (ID: MC11581) (^#^:44640633, Life Technologies, Grand Island, NY, USA) using LipofectamineRNAiMAX (^#^13778075, Life Technologies) as we previously described [22].

### Protein extraction, immunhistochemistry and western blotting

FFPE xenograft tumor sections were stained with hematoxylin and eosin for assessing microscopic morphology, necrosis and invasive pattern. Percentage of tumor necrosis was calculated from whole-section scans. Additional sections were immunostained following standard procedure as we previously described [44]. Primary antibodies were used against COX2, PAX8 and E/cadherin (Abcam, Abcam, Invitrogen), the streptavidin-biotin-peroxidase complex protocol using the LSAB+ Kit (DAKO, Carpenteria, CA, USA) was employed. Diaminobenzidine served as chromogen.

From fresh tissue samples total protein was extracted as we previously published [47]. Protein concentration was determined by BCA protein assay (Pierce Biotechnology, Rockford, IL, USA). Total protein was separated in 6% or 10% SDS– PAGE, transferred to a nitrocellulose membrane and incubated overnight with primary antibodies (MDGA1 antibody 1:200 dilution; FRAS1 (Cat^#^sc-98444, Santa Cruz Bio) antibody 1:100 dilution). For loading control membranes were stripped and re-probed for mouse anti-β-actin (1:1000, Cell Signalling Technology Inc). Anti-mouse, anti-rabbit-HRP conjugated IgGs were used as secondary antibodies (1:10000, W402B, W401B, Promega, Madison, WI, USA) or anti-goat –HRP from Santa Cruz Bio. Band intensities were quantified using Image J software (Bethesda, MD, USA).

### Statistical analysis

Differences were evaluated by Student's *T*-test with Welch's correction and by one-way ANOVA depending on group numbers. Bonferroni's corrections were applied for multiple comparison in pathway analysis. All data is presented as mean and standard error of the mean (SEM). *P* < 0.05 was considered significant.

## SUPPLEMENTARY MATERIALS FIGURES AND TABLES






