# Supplementary material for: Elucidating mechanisms of sunitinib resistance in renal cancer: an integrated pathological-molecular analysis
Source: Oncotarget. 2017 Dec 8;9(4):4661–74. doi: 10.18632/oncotarget.23163 (PMC5797004; doi:10.18632/oncotarget.23163)
Supplement: Supplementary file 1 [file oncotarget-09-4661-s001.pdf]

Elucidating mechanisms of sunitinib resistance in renal cancer: an integrated pathological-molecular analysis

SUPPLEMENTARY MATERIALS

miRNA and miRNA next generation sequencing MiRNA sequencing

Libraries were prepared using the Truseq Small RNA Library Sample prep kit (Illumina Inc., San Diego, CA, USA Cat #RS-200-0012). In brief, 1.5 ug of total RNA (RIN>7) was purified for RNA fragments less than 150bp using the PureLink miRNA isolation kit (Life Technologies, Carlsbad, CA, USA Cat#K1570-01). MiRNA was then precipitated using glycogen. Sample quality was assessed using the Bioanalyzer Small RNA kit (Agilent Technologies. Santa Clara, CA, USA Cat# 5067-1548). Next adapters were ligated to the RNA fragments' 3′ end using T4 RNA Ligase 2 Deletion mutant (Epicentre, Madison, WI, USA Cat# LR2D1132K) followed by ligating RNA adapters to the 5′ end of each fragment. cDNA synthesis was performed on miRNA using SuperScript II reverse transcriptase (Invitrogen, Carlsbad, CA, USA Cat #18064-014). Resulting adapter-ligated cDNA was amplified using 11 cycles of PCR. Pre-gel assessment of cDNA done using Bioanalyzer High Sensitivity DNA Kit (Agilent Technologies, Santa Clara, CA, USA Cat #5067-4626) to ensure the small RNA 140-160nt peak was present. Entire sample containing the amplified cDNA construct ran on 6% polyacrylamide gel for 60 min at 145V. Gel stained with 10000X SYBR Gold (Lifetech, Carlsbad, CA, USA Cat#S-11494) and visualized on transilluminator. Gel slice containing the 147 nt and 157 nt bands removed, DNA was eluted overnight and then filtered through a 5 µm filter tube. Libraries then concentrated with ethanol washing/precipitation steps and resuspended in Tris-HCl. Final libraries validated with Bioanalyzer High Sensitivity DNA Kit (Agilent Technologies, Santa Clara, CA, USA Cat #5067-4626) and quantified on the Eco Real-Time PCR Instrument (Illumina Inc., San Diego, CA, USA) using KAPA Illumina Library Quantification Kits (KAPA Biosciences, Woburn, MA, USA Cat# KK4835) according to the standard manufacturer's protocols. Single-read Cluster generation (Illumina Inc., San Diego, CA, USA Cat GD-402-4001) and rapid run sequencing of 1 x 51 cycles (Illumina Inc., San Diego, CA, USA Cat # FC-402-4002) was performed for the pooled libraries on the Illumina Hi-Seq 2500 platform (Illumina Inc., San Diego, CA, USA).

RNA sequencing

Libraries were prepared using the TruSeq Stranded mRNA Sample Prep Kit (Illumina, Cat #RS-122-2101). In brief, polyA containing mRNA molecules were purified from 4 ug of total RNA (RIN > 7) using poly-T oligos attached to magnetic beads. mRNA was then fragmented to approximately 300 bp during the polyA elution step. First strand cDNA synthesis was performed using SuperScript II reverse transcriptase (Invitrogen, Cat #18064-014) and random primers. Removing the RNA template and synthesizing the replacement strand, incorporating dUTP in place of dTTP, prepared double stranded cDNA, which was then used as template for library preparation. cDNA was then 3′end adenylated followed by ligation of the indexing adapters. Adapter-ligated DNA was enriched using 15 cycles of PCR. All purification and size selection steps were performed using AMPure XP SPRI beads (Beckman Coulter Genomics, Danvers, MA, USA Cat #A63881). Libraries were validated using the Agilent Bioanalyzer High Sensitivity DNA Kit (Agilent Technologies, Santa Clara, CA, USA Cat #5067-4626) and quantified on the Illumina Eco Real-Time PCR Instrument (Illumina Inc., San Diego, CA, USA) using KAPA Illumina Library Quantification Kits (KAPA Biosciences, Woburn, MA, USA Cat# KK4835) according to the standard manufacturer's protocols. Paired-end cluster generation (Illumina Inc., San Diego, CA, USA Cat #PE-401-3001) and sequencing of 2 x 101 cycles (Illumina Inc., San Diego, CA, USA Cat #FC-401-3001) was performed for all libraries on the Illumina Hi-Seq 2000/2500 platforms (Illumina Inc., San Diego, CA, USA).

Bioinformatical analysis of NGS data RNA sequencing

Paired end reads were trimmed to 75 bp to remove low quality sequence prior to alignment. Reads were aligned to the UCSC hg19 reference genome using tophat (v2.0.3), followed by transcript assembly and estimation of expression levels (fpkm) with cufflinks (v2.0.2). Reads from xenograft samples were first filtered to removed unambiguous mouse sequence using xenome(v1.0.1).

Differential expression between groups was determined using the cuffdiff module of cufflinks, providing fold change estimates, associated p-values and q-values. The cufdiff output was reviewed with the R package cummeRbund, to generate a variety of visualizations of the data and summaries of the most significant differently expressed events.

MiRNA sequencing

Reads were aligned to a database of mature RNA sequences (mirBase 20) using novoalign v2.08.02. The first 12 bases of the Illumina adapter (TGGAATTCTCGG) were supplied to novoalign for adapter trimming. The number of reads uniquely mapping to each mature RNA sequence were counted. Raw counts were normalized and transformed using the R package EdgeR and the voom transformation from the R package limma. Differential expression between groups was determined using the limma package.

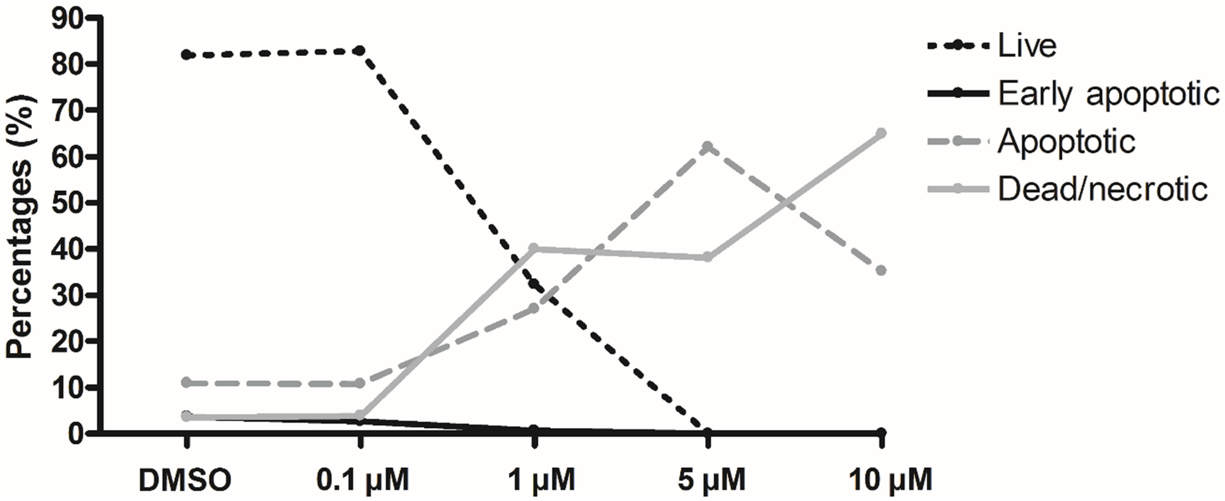

Supplementary Figure 1: Effect of different sunitinib concentration on apoptosis evaluated by AnnexinV & Propidium-Iodide flow cytometry.

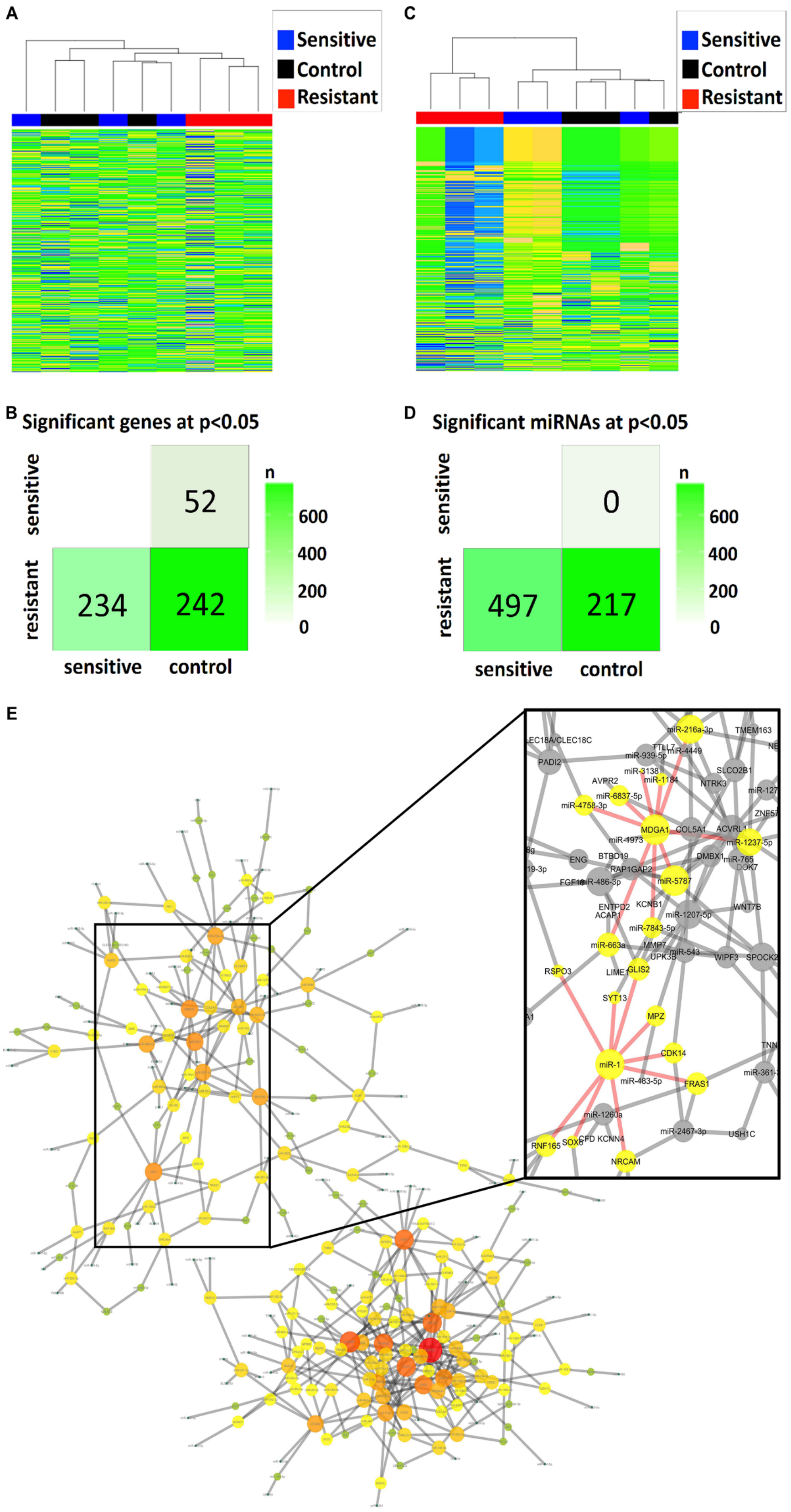

Supplementary Figure 2: Cluster analysis and number of differentially expressed genes between control, sensitive and resistant tumors based on mRNA analysis (**A**–**B**) and miRNA profiling (**C**–**D**). miRNA-target network analysis (**E**) comparing sunitinib-sensitive and resistant tumors. Each node (circle) represents a gene/miRNA, the connected lines represent interactions between molecules. HUBs are defined as the top 10% of the nodes with highest number of interaction. The analysis shows central involvement of miR-1/MDGA1 interactions (highlighted on the right in the rectangle).

Supplementary Table 1: BioFunction analysis of differentially expressed genes in sunitinib Sensitive xenograft compared to Control

Diseases or Functions Annotation	*p*-Value	Predicted Activation State	Activation z-score	#Molecules
proliferation of endothelial cells	2,14E-08	Decreased	-2,579	15
development of cardiovascular tissue	5,50E-08	Decreased	-2,579	16
development of cardiovascular system	5,99E-08	Decreased	-2,871	22
vasculogenesis	7,92E-08	Decreased	-2,903	19
development of blood vessel	9,63E-08	Decreased	-2,701	20
growth of epithelial tissue	3,61E-07	Decreased	-2,899	17
angiogenesis	2,93E-06	Decreased	-2,082	15
proliferation of connective tissue cells	4,39E-06	Decreased	-2,097	12
cell movement of endothelial cells	4,68E-06	Decreased	-2,536	13
migration of endothelial cells	1,01E-05	Decreased	-2,892	12
cell movement of neutrophils	3,31E-05	Decreased	-2,113	8
migration of cells	3,53E-05	Decreased	-2,359	32
migration of smooth muscle cells	6,52E-05	Decreased	-2,367	6
proliferation of smooth muscle cells	2,53E-04	Decreased	-2,39	7
cell movement of phagocytes	3,85E-04	Decreased	-2,563	10
quantity of Ca^{2+}	3,99E-04	Decreased	-2,393	10
cell movement of myeloid cells	4,12E-04	Decreased	-2,6	10
response of myeloid cells	4,98E-04	Decreased	-2,169	5
cell movement of leukocytes	6,53E-04	Decreased	-2,134	12
response of granulocytes	8,09E-04	Decreased	-2	4
immune response of leukocytes	2,94E-03	Decreased	-2,155	5
cellular homeostasis	6,14E-03	Decreased	-2,508	17
activation of leukocytes	1,02E-02	Decreased	-2,217	8
movement of vascular endothelial cells	1,46E-02	Decreased	-2,172	5

Supplementary Table 2: Pathway, BioFunction and Gene Onthology (GO) analysis of differentially expressed genes in sunitinib-resistant xenografts compared to sensitive tumors. See_Supplementary_Table 2

Supplementary Table 3: Pathway and BioFunction analysis of predicted targets of differentially expressed miRNAs between in vivo and in vitro experiments comparing sunitinib resistant states to sunitinib sensitive. See_Supplementary_Table 3

Supplementary Table 4: The most important nodes in the miRNA-target network

	Node name	Node degree
1	MEF2C	16
2	CXADR	12
3	SHC3	11
4	WNT5A	11
5	FGF1	11
6	PPAP2B	11
7	LRIG1	10
8	miR-1	9
9	MDGA1	9
10	DTNA	9
11	miR-5787	9
12	let-7i-5p	8
13	OTUB2	8
14	miR-1207-5p	8
15	SPOCK2	8
16	miR-216a-3p	8
17	miR-340-5p	8
18	miR-486-3p	8
19	FGF5	7
20	HAS3	7
21	ACVRL1	7
22	miR-1237-5p	7
23	RPS6KA2	7
24	miR-195-5p	7
25	miR-204-5p	7
26	miR-218-5p	7

Node degree shows the number of interacting partners.
